# Analysis of phosphorylation sites on autophagy proteins

**DOI:** 10.1007/s13238-015-0166-0

**Published:** 2015-06-17

**Authors:** Wenzhi Feng, Wenhao Zhang, Hui Wang, Lili Ma, Di Miao, Zexian Liu, Yu Xue, Haiteng Deng, Li Yu

**Affiliations:** PTN Program, College of Life Science, Peking University, Beijing, 100871 China; Center for Biomedical Analysis, Medical School, Tsinghua University, Beijing, 100084 China; Key Laboratory of Cardiovascular Remodeling and Function Research, Department of Cardiology, Qilu Hospital of Shandong University, Jinan, 250012 China; Department of Biomedical Engineering, College of Life Science and Technology, Huazhong University of Science and Technology, Wuhan, 430074 China; State Key Laboratory of Biomembrane and Membrane Biotechnology, Tsinghua University–Peking University Joint Center for Life Sciences, School of Life Sciences, Tsinghua University, Beijing, 100084 China

**Dear Editor,**

Autophagy is a lysosome-based degradation pathway, characterized by formation of a double-membrane vesicle named the autophagosome. During autophagy, various cargos are engulfed and delivered to lysosomes (or the vacuole in yeast) for degradation. It is an evolutionarily conserved pathway and plays important roles in various physiological and pathological conditions such as development, immunity, cancer and neuronal degeneration (Jiang and Mizushima, 2014; Martin et al., 2014).

Autophagosome formation is regulated by a set of autophagy-related (Atg) proteins. Phosphorylation plays an important role in the regulation of autophagy (McEwan and Dikic, 2011). Some Atg proteins, such as Atg1, Atg13, Atg29 and Atg31, have been reported to be phosphorylated (Kamada et al., 2000; Yeh et al., 2011; Mao et al., 2013), but so far there has been no systematic study of the phosphorylation of key autophagy proteins.

To analyze the phosphorylation sites of key autophagy proteins, we first purified Atg proteins, including Atg1, Atg3, Atg4, Atg6, Atg8, Atg15, Atg16, Atg17, Atg18, Atg27, Atg29 and Atg31, from yeast that had been grown in nutrient-rich conditions or starved for 1 h to induce autophagy. Proteins were digested with sequencing-grade trypsin and extracted from the gel. Peptides were subjected to several repeated rounds of tandem LC-MS/MS analysis in the data-dependent acquisition mode. The Mascot search engine was then used to compare the MS data against the *Saccharomyces cerevisiae* database using the Proteome Discoverer (Version 1.4) search algorithm. Each search result had no close second ranking protein contaminant. The corresponding MS/MS spectra for each of the tentatively assigned phosphorylation sites were manually verified and annotated for the sequence-informative b and y fragment ions. We also manually verified the intensity of each phospho-peptide and compared the phosphorylation ratio between cells in nutrient-rich medium and starvation medium. Through this process, we identified that many Atg proteins, including Atg1, Atg3, Atg4, Atg6, Atg15, Atg16, Atg18, Atg27, Atg29 and Atg31, are phosphorylated. More than 60 sites were identified on these proteins purified from both fed and starved cells (Tables 1 and S3).

**Table 1:** **Phosphorylation sites identified on yeast Atg proteins and mutagenesis screen**

Sites	Au L	Sites	Au L	Sites	Au L	Sites	Au L
Atg1 T226	+++	Atg1 S769	++−	Atg16 S50	+++	Atg29 T33	+++
Atg1 Y332	+++	Atg1 S783	++−	Atg18 S41, T56, S57	++−	Atg29 S43	+++
Atg1 S343	+++	Atg3 Y168, Y169	+++	Atg18 S140, S142, S146	++−	Atg29 S127	+++
Atg1 S351	+++	Atg3 Y172	+++	Atg18 S173	+++	Atg29 S187	+++
Atg1 S356	+++	Atg3 T177	+++	Atg18 S192, S195	++−	Atg31 S38, S40, T41, S44	+++
Atg1 S436	+++	Atg3 S230	+++	Atg18 S214	++−	Atg31 S116	+++
Atg1 S515	+++	Atg4 T483, S488	+++	Atg18 T234	+++	Atg31 S135	+++
Atg1 T590	+++	Atg6 S32, T37	+++	Atg18 S349	++−	Atg31 S143, S146	+++
Atg1 S621	++	Atg6 S231	+++	Atg18 T393	++−	Atg31 S153	++−
Atg1 S677	++	Atg15 T22, T24	+++	Atg27 T93	+++	Atg31 S174	+
Atg1 T685	+++	Atg16 S17, T21, S23	+++	Atg27 T211	+++	Atg31 S153, S195	+++

The identified phosphorylation sites were mutated to A and the autophagy activity of the mutants was assessed by monitoring the translocation of GFP-Atg8 into vacuoles. 100 cells were assessed in a blind fashion and quantified. The autophagy level ratio (Au L) is the qualitative assessments of mutant autophagy activity compared to WT activity

Next, we mutated each individual phosphorylation site to alanine (A). We also generated some mutants in which combinations of different sites are mutated. We found that most single phosphorylation site mutations have no effect on autophagy, except a few sites on Atg1 which have been reported previously (Yeh et al., 2011), and one site on Atg31 (S174, Table 1).

We identified 11 phosphorylation sites on Atg31. There was no significant change in the phosphorylation level at those sites when autophagy was induced (Fig. 1A) (ratio of IR in starvation medium/full medium >2 or <0.5). So are the phosphorylation sites on Atg3, Atg4, Atg6, Atg15, Atg16, Atg27 and Atg29, except one site, T177 phosphorylation on Atg3, appeared in starved cells. For other Atg proteins we tested, starvation increased the phosphorylation level. For example, 13 phosphorylation sites were identified on Atg1: T226, Y332, S343, S351, S356, S436, S515, T590, S621, S677, T685, S769 and S783 (Fig. 1B). Of these, T226, Y332, S343, S351, S356, S436 and T685 were different from the Atg1 phosphorylation sites reported before (Yeh et al., 2011). Y332 and T685 were new sites that have never been identified in previous studies of yeast phospho-proteomics. Phosphorylation of S343, S515, S685 and S769 was only detected under starvation conditions, while phosphorylation of Y332, S351 and S677 was only detected in cells cultured in nutrient-rich medium. When we compared the relative levels of phosphorylated Atg1 peptides under nutrient-rich conditions and starvation conditions, we found that most of the sites identified from the mass spectrometry analysis, including T226, S343, S356, S515, S621, T685 and S769, were phosphorylated at significantly higher levels (ratio of IR in starvation medium/IR in full medium >2) when autophagy occurs (Fig. 1D). Similarly, we identified 13 phosphorylation sites on Atg18 including 10 (S41, T56, S57, S140, S142, S146, S192, S195, S214 and T234) that are phosphorylated in fed cells and 9 (S140, S142, S146, S173, S195, S214, T234, S349 and T393) that are phosphorylated after induction of autophagy (Fig. 1C). The level of T234 phosphorylation increased significantly when autophagy was induced and three new phosphorylated sites appeared (S173, S349 and T393; Fig. 1E).

**Figure 1:**
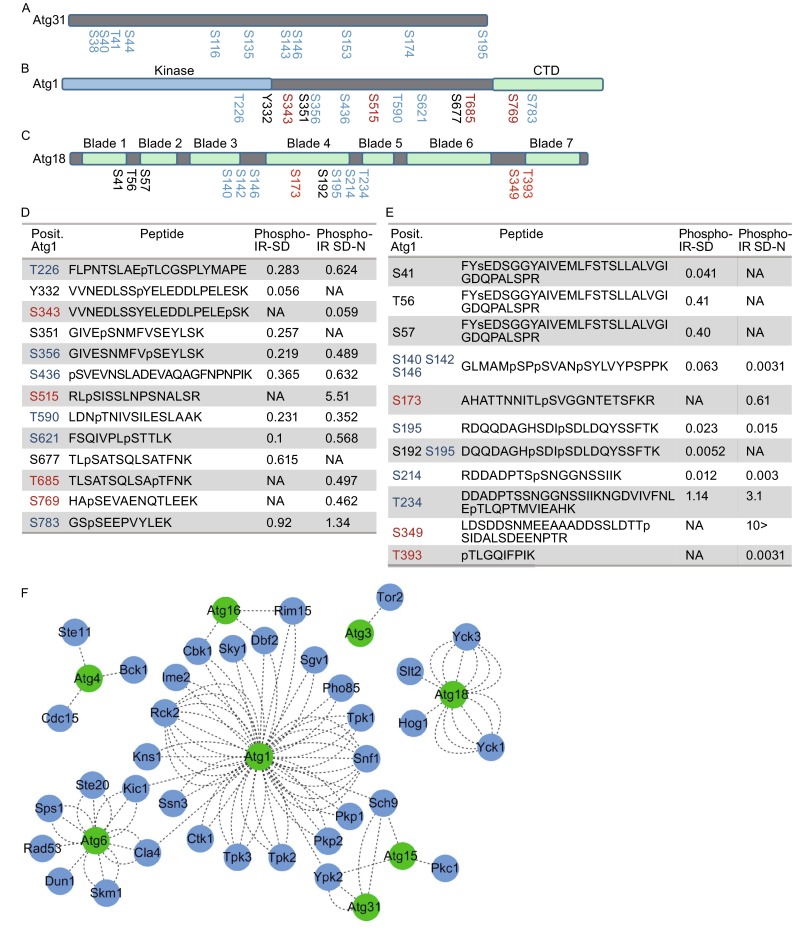
**Phosphorylation status of Atg proteins under nutrient-rich and starvation conditions**. (A–C) Schematic diagrams of the Atg31, Atg1 and Atg18 proteins showing the phosphorylation sites identified by MS. Blue, sites that are phosphorylated under both nutrient rich and starvation conditions; red, sites that are phosphorylated only under starvation conditions; black, sites that are phosphorylated only under nutrient-rich conditions. (D) MS1 intensity ratio (IR) of phosphorylated Atg1 peptides in nutrient-rich and starvation conditions. NA, no phospho-peptide detected. (E) MS1 intensity ratio (IR) of phosphorylated Atg18 peptides in nutrient-rich and starvation conditions. (F) Network of Atg proteins and the corresponding kinases predicted by iGPS

Finally, we predicted the kinase families responsible for phosphorylating the sites we identified (Xue et al., 2011) (Fig. 1F). We found that Atg1 can be phosphorylated by many important kinases: AMPK (Snf1), PKA (Tpk1/2/3), mitochondrial protein kinases (Pkp1/2), protein kinases involved in stress (Rck2, Sch9, Rim15 and Dbf2), protein kinases of the PAK/Ste20 family (Kic1, Cla4), kinases involved in transcription (Kns1, Sky1, Ssn3, Sgv1, Ctk1 and Pho85), kinases related to the cell integrity pathway (Ypk2, Cbk1), and others. Of these kinases, AMPK and PKA have previously been reported as mediating signals upstream of Atg1 (Stephan et al., 2009; Kim et al., 2011). The other predicted kinases suggest new signal pathways which may regulate Atg1 function or autophagy. Atg18 is predicted to be phosphorylated by the mitogen-activated protein kinases (MAPK) Hog1 and Slt2, yeast casein kinase homolog Yck1, and the vacuolar casein kinase I (CKI) Yck3. Yck3 is a palmitoylated vacuolar membrane-localized casein kinase I isoform that regulates vacuole fusion and fission. Hog1 is involved in osmoregulation. Atg4 is predicted to be phosphorylated by Ste11, Bck1 and Cdc15. Atg6 is predicted to be phosphorylated by Kic1, Cla4, Skm1, Dun1, Rad53, Sps1 and Ste20. Atg16 is predicted to be phosphorylated by Cbk1, Dbf2 and Rim15. The predicted kinases of Atg15 are Ypk2, Sch9 and Pkc1. And Atg31 is predicted to be phosphorylated by Ypk2 and Sch9.

In summary, we studied the phosphorylation of key Atg proteins and found that many of them were phosphorylated. Furthermore, we performed site-directed mutagenesis to check whether the phosphorylated sites have an effect on autophagy activity. Although most of the single mutations had no effect, we cannot exclude the possibility that different phosphorylation sites may combine together to play a role in autophagy. Finally, we predicted the kinases which may mediate phosphorylation of these Atg proteins. We hope our study provides useful information to researchers who study the regulation of autophagy.

## Electronic supplementary material

Supplementary material 1 (DOCX 20 kb)
