# Supplementary material for: Analysis of phosphorylation sites on autophagy proteins
Source: Protein Cell. 2015 Jun 17;6(9):698–701. doi: 10.1007/s13238-015-0166-0 (PMC4537473; doi:10.1007/s13238-015-0166-0)
Supplement: Supplementary file 1 — Supplementary material 1 (DOCX 20 kb) [file 13238_2015_166_MOESM1_ESM.docx]

**Supplemental Table1**

Yeast strains used in this sutdy.

| Name | Genotype | Reference |
| --- | --- | --- |
| BY4741 | *MATa his3Δ1 leu2Δ met15Δ ura3Δ* | This study |
| ScLY1 | BY4741, *pRS316[GFP-ATG8]* | This study |
| ScLY2 | BY4741, *pRS315[GFP-ATG8]* | This study |
| ScLY3 | BY4741, *YEPlac181 [Gal1-GST-ATG31]* | This study |
| ScLY4 | BY4741, *atg31∆::kanMX6* | This study |
| ScLY6 | ScLY4, *YEPlac181 [Gal1-GST-ATG31]* | This study |
| ScLY27 | BY4741, *atg1∆::kanMX6, YEPlac181 [Gal1-GST-ATG1]* | This study |
| ScLY28 | BY4741, *atg3∆::kanMX6, YEPlac181 [Gal1-GST-ATG3]* | This study |
| ScLY29 | BY4741, *atg4∆::kanMX6, YEPlac181 [Gal1-GST-ATG4]* | This study |
| ScLY30 | BY4741, *atg6∆::kanMX6, YEPlac181 [Gal1-GST-ATG6]* | This study |
| ScLY31 | BY4741, *atg8∆::kanMX6, YEPlac181 [Gal1-GST-ATG8]* | This study |
| ScLY32 | BY4741, *atg15∆::kanMX6, YEPlac181 [Gal1-GST-ATG15]* | This study |
| ScLY33 | BY4741, *atg16∆::kanMX6, YEPlac181 [Gal1-GST-ATG16]* | This study |
| ScLY34 | BY4741, *atg17∆::kanMX6, YEPlac181 [Gal1-GST-ATG17]* | This study |
| ScLY35 | BY4741, *atg18∆::kanMX6, YEPlac181 [Gal1-GST-ATG18]* | This study |
| ScLY36 | BY4741, *atg27∆::kanMX6, YEPlac181 [Gal1-GST-ATG27]* | This study |
| ScLY37 | BY4741, *atg29∆::kanMX6, YEPlac181 [Gal1-GST-ATG29]* | This study |
| ScLY38 | BY4741, *atg31∆::kanMX6, YEPlac181 [Gal1-GST-ATG31]* | This study |

**Supplemental table 2**

Mutagenesis primers

| Atg1 primer |  |
| --- | --- |
| T226A | ACGTCATTAGCAGAAgCTCTTTGTGGCTCACCA |
|  | TGGTGAGCCACAAAGAGcTTCTGCTAATGACGT |
| Y332A | GAAGACTTGTCTTCTgcTGAATTGGAAGATGA |
|  | TCATCTTCCAATTCAgcAGAAGACAAGTCTTC |
| S343A | TTACCTGAGTTAGAAgCCAAATCAAAAGGTATT |
|  | AATACCTTTTGATTTGGcTTCTAACTCAGGTAA |
| S351A | AAAGGTATTGTAGAAgcTAATATGTTCGTTTCT |
|  | AGAAACGAACATATTAgcTTCTACAATACCTTT |
| S356A | AATATGTTCGTTgCTGAGTATTTATCTAAACAG |
|  | CTGTTTAGATAAATACTCAGcAACGAACATATT |
| S436A | GTGGTAGAGAAGAAAgCGGTTGAAGTTAATTCA |
|  | TGAATTAACTTCAACCGcTTTCTTCTCTACCAC |
| S515A | GTGGATAGACGCTTGgCTATATCCTCGCTGAAT |
|  | ATTCAGCGAGGATATAGcCAAGCGTCTATCCAC |
| T590A | CTGAAATTAGATAATgCTAATATCGTTAGTATT |
|  | AATACTAACGATATTAGcATTATCTAATTTCAG |
| S621,T622, T623A | TCTCAAATTGTTCCATTAgCAgCAgCATTAAAAGGCATGGCTAAC |
|  | GTTAGCCATGCCTTTTAATGcTGcTGcTAATGGAACAATTTGAGA |
| S677A | AAGGGTAGAACTTTAgCTGCCACATCTCAGTTG |
|  | CAACTGAGATGTGGCAGcTAAAGTTCTACCCTT |
| T685A | TCTCAGTTGAGTGCAgCTTTCAATAAACTACCA |
|  | TGGTAGTTTATTGAAAGcTGCACTCAACTGAGA |
| S769A | AGATTCAAGCATGCTgCTGAGGTAGCTGAAAAT |
|  | ATTTTCAGCTACCTCAGcAGCATGCTTGAATCT |
| S783A | GAAGAAAAAGGTAGTgCGGAAGAGCCAGTATAT |
|  | ATATACTGGCTCTTCCGcACTACCTTTTTCTTC |
| Atg3 |  |
| Y168, Y169A | ATGGCGCAAGAAAGGgcTgcCGACCTTTATATTGCG |
|  | CGCAATATAAAGGTCGgcAgcCCTTTCTTGCGCCAT |
| Y172A | AGGTATTACGACCTTgcTATTGCGTACTCGACA |
|  | TGTCGAGTACGCAATAgcAAGGTCGTAATACCT |
| T177A | TATATTGCGTACTCGgCATCTTATAGGGTCCCT |
|  | AGGGACCCTATAAGATGcCGAGTACGCAATATA |
| S230A | TCAGTGTTATCTGTTgCCATTCATCCATGTAAG |
|  | CTTACATGGATGAATGGcAACAGATAACACTGA |
| Atg4 |  |
| T483A | GTCCTTGTAGAGAAGGAAgCGGTAGGTATTCAC |
|  | GTGAATACCTACCGcTTCCTTCTCTACAAGGAC |
| S488A | GTAGGTATTCACgcTCCTATTGATGAAAAA |
|  | TTTTTCATCAATAGGAgcGTGAATACCTAC |
| Atg6 |  |
| S32, T37A | ATTTGCTACTTTCAAATAATgCAATTATCACTGCAgCGAATGAAAATGTCATCAGCAAC |
|  | GTTGCTGATGACATTTTCATTCGcTGCAGTGATAATTGcATTATTTGAAAGTAGCAAAT |
| S231A | AAAGAAAAGCAGTATgCCCATAATCTTTCGGAA |
|  | TTCCGAAAGATTATGGGcATACTGCTTTTCTTT |
| Atg15 |  |
| T22, T24A | CTAGGATGCATTCTAgCGCTTgCAGTGCTCTGCCTTATT |
|  | AATAAGGCAGAGCACTGcAAGCGcTAGAATGCATCCTAG |
| Atg16 |  |
| S17,T21, S23A | AAAGCAAAGGAGGAAAGAgcCAATCCACAAgCAGATgcCATGGATGATTTGTTAATT |
|  | AATTAACAAATCATCCATGgcATCTGcTTGTGGATTGgcTCTTTCCTCCTTTGCTTT |
| S50A | TTGTTTCAAGATAATgcTGGCGCCATTGGTGGC |
|  | GCCACCAATGGCGCCAgcATTATCTTGAAACAA |
| Atg18 |  |
| S41A | TTCGGAAAATTTTATgCAGAGGACAGTGGGGGC |
|  | GCCCCCACTGTCCTCTGcATAAAATTTTCCGAA |
| T56, S57A | GTCGAGATGTTGTTCTCCgCCgCGTTACTAGCCCTCGTTGGG |
|  | CCCAACGAGGGCTAGTAACGcGGcGGAGAACAACATCTCGAC |
| S140,S142, S146A | CCACGTGGCCTTATGGCTATGgCTCCTgCGGTAGCCAACgcCTATTTAGTGTATCCATCACCACCA |
|  | TGGTGGTGATGGATACACTAAATAGgcGTTGGCTACCGcAGGAGcCATAGCCATAAGGCCACGTGG |
| S173A | AACAATATCACATTGgCAGTTGGTGGCAACACA |
|  | TGTGTTGCCACCAACTGcCAATGTGATATTGTT |
| S192A, S195A | GATCAGCAAGATGCTGGCCATgcTGACATTgcCGACTTGGATCAGTATTCGAGC |
|  | GCTCGAATACTGATCCAAGTCGgcAATGTCAgcATGGCCAGCATCTTGCTGATC |
| S214A | GCGGATCCAACAAGCgcTAACGGCGGTAACAGC |
|  | GCTGTTACCGCCGTTAgcGCTTGTTGGATCCGC |
| T234A | GTATTCAACTTGGAAgCATTACAGCCAACCATG |
|  | CATGGTTGGCTGTAATGcTTCCAAGTTGAATAC |
| S349A | TCGCTCGATACCACCgcTATCGATGCGCTGAGT |
|  | ACTCAGCGCATCGATAgcGGTGGTATCGAGCGA |
| T393A | CGAAGAGCTGCCAGAgCATTGGGTCAGATTTTC |
|  | GAAAATCTGACCCAATGcTCTGGCAGCTCTTCG |
| Atg27 |  |
| T93 | AAGGATGCTATCACCgCTCAAATTATAGATTTT |
|  | AAAATCTATAATTTGAGcGGTGATAGCATCCTT |
| T211 | TACGCTCTACTGTTTgCATTGATATACCTGATG |
|  | CATCAGGTATATCAATGcAAACAGTAGAGCGTA |
| Atg29 |  |
| T33A | TTTGAGTGGAATGGAgCAAAGGAGCGACAGCTT |
|  | AAGCTGTCGCTCCTTTGcTCCATTCCACTCAAA |
| S43A | GCTTTGGACAATGGTAgCAAATTTGAATTATTC |
|  | GAATAATTCAAATTTGcTACCATTGTCCAAAGC |
| S127A | TAAATGTATCCGCAgcCCCACTGACCACGGAAA |
|  | TTTCCGTGGTCAGTGGGgcTGCGGATACATTTA |
| S187A | GAAATGGAGTGCGGTgcTTCAGATGACGATTTAT |
|  | ATAAATCGTCATCTGAAgcACCGCACTCCATTTC |
| Atg31 |  |
| S38, S40, T41, S44A | ATCAACCAGCATACAATAATGAAgCAAAGgcCgCGGACGGAgcTGATTATGCAATGTTTCCCACTAACATTAAG |
|  | CTTAATGTTAGTGGGAAACATTGCATAATCAgcTCCGTCCGcGgcCTTTGcTTCATTATTGTATGCTGGTTGAT |
| S116A | ATGAGCTTTTGTCACATAGGACAAATGCTCTCAGCTTGGAGG |
|  | CCTCCAAGCTGAGAGCATTTGTCCTATGTGACAAAAGCTCAT |
| S135A | TTATCATCACATGGTGATGATAAAGCTAATGATGAAGAGGAAGAAC |
|  | GTTCTTCCTCTTCATCATTAGCTTTATCATCACCATGTGATGATAA |
| S143A,S146A | ATCTAATGATGAAGAGGAAGAACTTGCCGTTGACGCTGATAGATTCAGAGTGGACTC |
|  | GAGTCCACTCTGAATCTATCAGCGTCAACGGCAAGTTCTTCCTCTTCATCATTAGAT |
| S153A | ATAGATTCAGAGTGGACGCGGACATTGAGCTCGAT |
|  | ATCGAGCTCAATGTCCGCGTCCACTCTGAATCTAT |
| S174A | TCTGTCTCCTTTTCTTCGCGATTTGGCTTTGAATGACTTAATTAAACTCTAC |
|  | GTAGAGTTTAATTAAGTCATTCAAAGCCAAATCGCGAAGAAAAGGAGACAGA |
| s195A | TGAACAATTACAAATGCTCTCCAATGCCGTATGAAAGCAAAAATGA |
|  | TCATTTTTGCTTTCATACGGCATTGGAGAGCATTTGTAATTGTTCA |

**Supplemental table 3**

Phosphorylation sites identified by mass spectrum in full medium and starvation medium.

| **Protein name** | **Phospho-sites in full medium** | **Phospho-sites in starvation medium** |
| --- | --- | --- |
| Atg1 | T226, Y332, S351, S356, S436, T590, S621, S677, S783 | T226, S343, S356, S436, S515, T590, S621, T685, S769, S783. |
| Atg3 | Y168, Y169, Y172, S230 | Y168, Y169, Y172, T177, S230 |
| Atg4 | T483, S488 | T483, S488 |
| Atg6 | S32, T37, S231 | S32, T37, S231 |
| Atg15 | T22, T24 | T22, T24 |
| Atg16 | S17, T21, S23, S50 | S17, T21, S23, S50 |
| Atg18 | S41, T56, S57, S140, S142, S146, S192, S195, S214, T234 | S140, S142, S146, S173, S195, S214, T234, S349, T393 |
| Atg27 | T93, T211 | T93, T211 |
| Atg29 | T33, S43, S127, S187 | T33, S43, S127, S187 |
| Atg31 | S38, S40, T41, S44, S116, S135, S143, S146, S153, S174, S195 | S38, S40, T41, S44, S116, S135, S143, S146, S153, S174, S195 |
